# Local General Practitioner–Parish Minister Networks for Existential Care in Danish Primary Care—What Did We Learn? A Ricoeur-Inspired Focus Group Study

**DOI:** 10.3390/ijerph23020175

**Published:** 2026-01-30

**Authors:** Lone Vesterdal, Inger Uldall Juhl, Charlotte Simonÿ, Ricko Damberg Nissen, Niels Christian Hvidt

**Affiliations:** 1 Research Unit of User Perspectives and Community-Based Interventions, Institute of Public Health, Faculty of Health Sciences, University of Southern Denmark, 5230 Odense, Denmark; 2 Research Unit of General Practice, Institute of Public Health, Faculty of Health Sciences, University of Southern Denmark, 5230 Odense, Denmark; 3 The Research and Implementation Unit PROgrez, Department of Physiotherapy and Occupational Therapy, Central and West Zealand Hospital, 4600 Køge, Denmark; 4 Department of Regional Health Research, University of Southern Denmark, 5230 Odense, Denmark; 5 Forensic Mental Health Research Unit Middelfart (RFM), Department of Regional Health Research, Faculty of Health Science, University of Southern Denmark, 5230 Odense, Denmark; 6 Psychiatric Dept. Middelfart, Mental Health Services in the Region of Southern Denmark, 5230 Odense, Denmark

**Keywords:** existential support, spiritual care, focus group interview, Ricoeur-inspired analysis, local interdisciplinary network, participatory action research

## Abstract

Background: Local networks between General Practitioners (GPs) and Parish Ministers (PMs) have been piloted in Denmark to address the lack of collaboration between the two groups in order to strengthen existential and spiritual support in primary care. Evidence on how such collaborations are experienced by practitioners is limited. Aim: The objective was to explore the experience of GPs and PMs participating in locally established interdisciplinary networks. Design and Methods: Within a Ricoeur-inspired phenomenological hermeneutical framework, we conducted five focus group interviews with five GPs and nine PMs from four Danish localities engaged in a step-by-step, participant-validated networking manual. Data was analyzed using a three-level process, including naïve reading, structural analysis, and critical interpretation and discussion. Results: Participants described the collaboration as an educational, relationship-building process that required time and trust. Four themes emerged: (1) sharpening professional identity (GPs reframed limits of “fixing,” and PMs broadened pastoral scope); (2) building relationships (mutual prejudices surfaced and were dismantled; in-person meetings were pivotal); (3) serving the patient’s perspective better (PMs offered a non-clinical space for existential issues; early patient involvement energized groups); and (4) envisioning PMs’ role in primary care (promise of complementarity vs. value of remaining outside formal health system documentation). Conclusions: Locally grown GP–PM networks can reframe practice for both professions and open a pragmatic pathway for addressing patients’ existential concerns. Relationship-building and early, appropriate patient inclusion appear central to momentum. Further research should examine patient outcomes and feasible models for collaboration that preserve confidentiality and role clarity.

## 1. Introduction

In the everyday reality of primary care, General Practitioners (GPs) often consult patients whose suffering cannot be measured or fixed. These encounters reveal a dimension of care that stretches beyond the biomedical frame—a dimension where questions of meaning, loss, and hope emerge. For many GPs, this space feels unfamiliar, even unsettling, as their professional identity is shaped by the imperative to diagnose, treat, and resolve [1,2,3,4]. Yet, patients’ existential concerns persist, calling for a response that is not only clinical but deeply human.

Pastoral caregivers, such as Parish Ministers (PMs), inhabit this existential space as part of their vocation (we use the title Parish Minister to designate the difference between hospital chaplains and parish minister in the local community). Their practice is grounded in presence, listening, and accompanying individuals through life crises without the expectation of resolution. When these two worlds meet—the clinical and the pastoral—new possibilities arise for patient care and for the professionals themselves. International experiences in Scotland, England, and the USA have shown that such encounters can transform practice: they foster holistic care, dismantle prejudices, and create room for reframing what it means to support a patient [5,6,7,8,9,10,11].

In Denmark, similar initiatives have begun to take root. Local networks between GPs and PMs have been piloted to strengthen existential and spiritual support in primary care [12,13]. In this article, we define spiritual care as the support of patients’ spiritual and existential needs and challenges [14,15].

Since 2022, a research project initiated by Inger Uldall Juhl og Lone Vesterdal has striven to design and investigate collaboration between GPs and PMs [13,16,17,18,19]. This early initiative involves seven GPs and six PMs and has served as foundational model for the development of similar community-based collaboration efforts across Denmark [17].

Six clusters across different regions in Denmark were established, each including GPs and PMs. As such, 35 GPs and PMs were involved in the initiative. A manual was developed to guide the newly established collaboration [12]. These efforts resonate with broader healthcare reforms that emphasize continuity, local anchoring, and interdisciplinary collaboration [20,21,22,23]. Yet, the process is not without challenges. How do two professions, each with their own language, traditions, and boundaries, learn to collaborate meaningfully? How can trust grow in a system marked by time pressure and documentation requirements? And what does it mean to offer existential care in a pluralistic society?

These questions are not merely organizational—they touch the lived experience of professionals and patients alike. They invite us to explore how collaboration is felt, understood, and enacted in practice. Against this backdrop, our research asks the following:

How do General Practitioners and Parish Ministers experience participating in locally established interdisciplinary networks aimed at strengthening existential and spiritual support in Danish primary care?

## 2. Materials and Methods

### 2.1. Design and Setting

This study employed a phenomenological hermeneutic approach inspired by the philosophy of Paul Ricoeur [24,25,26,27].

Six interdisciplinary groups were formed in different regions of Denmark. In this study, the groups are named Groups 1, 2, 3, 4, 5, and 6 for data protection.

Supervision processes led by the two project leaders took place throughout autumn 2024 and early spring 2025.

During this period, Groups 5 and 6 withdrew from the project. In Group 5, the nine participants reviewed the manual but chose to focus solely on fostering social connections between the professional groups, opting not to involve patients directly. In Group 6, a disagreement arose at the first meeting between the 12 participants.

The GPs had been invited by the local diocese leadership, and the collaboration did not proceed further. During the meeting in Group 6, the GPs felt that the local diocese leadership “had it all arranged in advance,” leaving them with no real opportunity to contribute as co-creators. They perceived their role as merely endorsing a pre-defined project, and for this reason, they chose to withdraw.

### 2.2. Participant Inclusion/Exclusion

The six test groups were included in the validation process because they had contacted Juhl and Vesterdal to express interest in the project. This recruitment method resulted in groups with strong commitment as they had not only volunteered but themselves sought out the project. The aim was from the beginning not to include all GPs and PMs in Denmark, as the process required long-term engagement, supervision, and guidance. For this reason, we prioritized groups that demonstrated strong commitment and chose a number that allowed for close supervision throughout the project.

### 2.3. Data Generation (Focus Group Interviews)

Drawing on Ricoeur’s theory of narrative, dialog, and the threefold mimesis [25,26,28], we conducted focus group interviews (FGIs) as a means of generating rich, collective narratives of lived experience. Our focus was to explore GPs’ and PMs’ experiences, interpretations, and reflections on the collaborative initiative concerning their professional practice.

Five FGIs with a total of 14 participants (5 GPs and 9 PMs) were held, one in October 2024 and four in January 2025. One was conducted in person with three GPs and six PMs (3 October 2024). The remaining four FGIs were held online via Teams (17 January 2025, 20 January 2025, 20 January 2025, 22 January 2025) with local grounded groups across Denmark, from different areas. The participants in the interview in October 2024 came from different regions in Denmark. They did not know each other before the interview. The interviews held in January 2025 were performed in each geographical group. In these interviews, they knew each other very well because they had collaborated for at least 12 months. This combination of interviews resulted in a variety of perspectives. Especially in the October interview, they could not take each other’s understanding for granted [28,29].

A semi-structured interview guide was developed and discussed by the research group [30,31].

We determined that a pilot test would be unnecessary, based on our regular contact with the groups and our insight into their challenges. As prescribed by Simonÿ et al. [28], the focus group interviews were designed to establish a hermeneutic environment, encourage narrative sharing, promote ethical engagement, and embrace ambiguity and complexity. Within this frame, we used the following open-ended questions to conduct the FGIs:

Did the experience of collaborating in patient care encourage reflections on daily practice and perceptions of the other professional group? If so, what were these reflections? Could the collaborative initiative support patient care, and if so, how? What are the hopes and expectations for future collaboration?

The interviews lasted between 30 and 40 min. They are recorded and transcribed verbatim by the first author. All data were securely stored on the S4 safe server at the University of Southern Denmark (SDU).

### 2.4. Analysis

We sought to explore the significance of local, interdisciplinary collaboration for participants—and to convey their experiences by listening to the stories they told. Through these stories, we sought to understand how participants perceive their reality as professionals, and what kinds of meaning the collaboration held for them.

**Figure 1:**
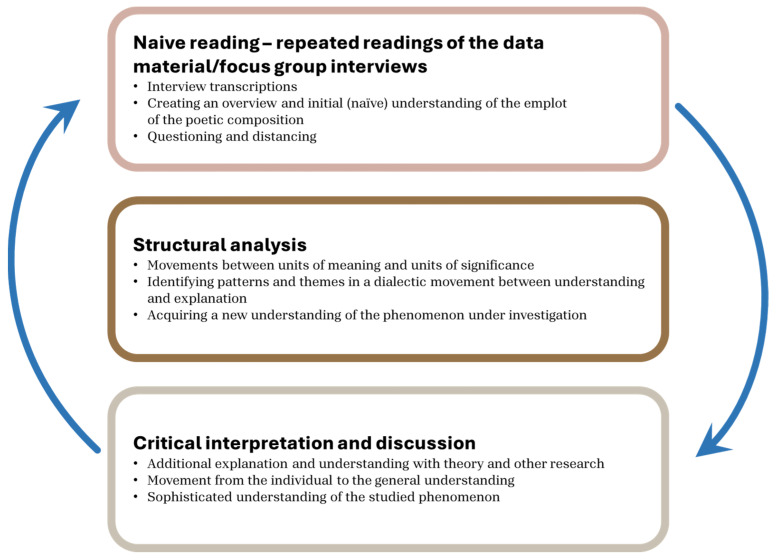
The Ricoeur-inspired interpretation includes dialectical movements between the initial naïve reading, the structural analysis, and the critical interpretation and discussion. The interpretation follows a hermeneutical spiral and moves from a naïve to a sophisticated understanding [24,28,32,34]. Thus, this reveals a new understanding, ready to be applied in practice.

As described by Simonÿ et al., we conducted a Ricoeur-inspired analysis [28,32,33]. This included a three-level interpretive process including naïve reading, structural analysis, and critical interpretation and discussion. In the naïve reading, we listened to the recorded interviews and read the transcripts with an open mind, setting aside preconceptions. Hereby, we formed an early sense of what the participants were talking about. Following this, we conducted a structural analysis [28,32,33]. While moving back and forth between understanding and explanation, we identified what the text was about. This led to four themes. Through this hermeneutic spiral, we sought to move from an initial understanding of the text to a deeper appropriation of the meaning embedded in the participants’ shared narratives. Through this procedure, we were able to thereby illuminate GPs’ and PMs’ experiences of being-in-the-world within the context of the new collaborative network. The themes are described in Section 3, followed by critical interpretation and discussion in Section 4. The critical interpretation and discussion are the final level in the interpretation, which serves to bring the interpretation to its conclusion and move findings from the individual to the general [24,28,32]. Figure 1 shows the process of the three-level interpretation:

### 2.5. Author’s Note

All citations have been translated from Danish and lightly edited to ensure clarity and readability in English, while preserving the original tone and intent. The translations have been read and approved by the authors.

Two of the researchers in this study possessed prior contextual knowledge, as they are themselves a GP (Inger Uldall Juhl) and a PM (Lone Vesterdal) in Kolding and actively engaged in the research project. Their dual role may have introduced a potential bias, in that other participants could have been less inclined to adopt a critical or negative stance. Nevertheless, the observed openness, dynamic exchanges, and free tone within the FGIs indicate that these circumstances did not compromise the validity of the study.

### 2.6. Ethical Statement and Project Review

Verbal information was provided individually to all participants, explaining that (a) the interviews form part of a research project; (b) the data are stored on a secure server and anonymized; (c) the data will be used exclusively for research purposes; and (d) participants may withdraw at any time without consequence. Oral consent was obtained accordingly. This procedure complies with Danish Health Act §10.

Prior to implementation, the study design and questionnaire were reviewed by the University of Southern Denmark, Research & Innovation Organization (SDU RIO, journal number 11.585). The study was conducted in full compliance with Danish law on the procession of personal data as well as the Helsinki Declaration [35].

## 3. Results

The naïve reading reflected that it was important for participants to establish, shape, and develop the collaboration in a way that felt meaningful to them and adapted to the local context. They emphasized uniqueness in their collaboration, facilitating benefits for patients and themselves. Time was an essential part of the process. Seemingly, the collaboration required time and mutual trust. Participants described it as a relationship-building process, during which mutual prejudices surfaced and were dismantled.

Participants expressed surprise at discovering how the other profession practiced. This process of getting to know each other was both enriching and inspiring—personally and professionally—and it helped sharpen their own practice. They described the collaboration as an educational journey that brought two professional groups together. While there was no resistance to including PMs in primary care, some participants reflected on whether the PM’s role should be formally recognized within the healthcare system or remain external to it.

From the structural analysis, four overarching themes were identified, capturing the core dimensions of participants’ experiences of the collaborative initiative. Together, the themes reflect how the collaboration shaped professional identity, required deliberate relationship-building, enhanced patient care—particularly regarding existential concerns—and prompted reflections on the future role of PMs within primary care.

The four themes were:

1. Sharpening Professional Identity;
2. Building Relationships;
3. Better Serving the Patients’ Perspective;
4. Envisioning PMs’ Role in Primary Care.

The themes are presented below and illustrated by selected quotations from the focus group interviews.

### 3.1. Theme 1: Sharpening Professional Identity

The first theme concerns how participation in the collaboration prompted both GPs and PMs to reflect on and re-evaluate their own professional identities and practices. Through encountering the other profession, participants described gaining a clearer sense of their own professional boundaries, roles, and core tasks—almost as if they were able to view themselves from the outside.

One GP reflected that getting to know the PMs helped them clarify professional limitations. They stated the following:

“I’ve been reminded that I don’t have to fix everything. We fix, fix, fix all the time—and if we can’t fix something, we try again, schedule another consultation, and keep trying. The tempo needs to be high. But it’s okay to say: ‘I can’t fix this.’ It’s okay just being with the patient in that difficult situation without solving it. That’s been a real relief for me” (GP 2, Group 2).

In line with this, the interviews covered how GPs experienced themselves as better supporting patients in accepting and dealing with existential matters while participating in the collaborative initiative. A GP disclosed how the initiative has “expanded her horizon”, and that she thinks she has become a better doctor by communicating with another professional group. Participation in the interdisciplinary network thus led to renewed, clarified perceptions of the core tasks within their own practice. GPs and PMs described that being brought together in the interdisciplinary initiative improved their respective practices. The GPs shared experiences of inadequacy in certain patient encounters with existential issues, and the collaborative initiative provided them with support in patient care. The PMs, on their part, elaborated enthusiastically on how the collaboration has brought new challenges into their pastoral care. One of them said the following:

“When you’ve been a PM for more than 20 years, you know loss and bereavement. You quickly sense where the pain lies. But the people referred to by the GP come with all kinds of life crises, and I really must stay sharp, be attentive, and figure out how to meet that. It’s demanding—but also deeply rewarding. It brings new energy to my work, and I feel I’m growing as a professional” (PM 5, Group 2).

Participation also led to a sharpened focus on their pastoral identity and possible pitfalls. They emphasized their professional limits in being theologians and pastors, and not psychologists.

The theme highlights how the interdisciplinary initiative paved the way for improved patient care from a holistic perspective. In a remarkable way, it supported the GPs to be with the patients in powerlessness instead of trying to fix what cannot be fixed. For both GPs and PMs, the collaboration thus led to professional self-confrontation resulting in a sharpened professional identity.

While these reflections primarily concerned the participants’ own professional self-understanding, they were closely intertwined with the relationships that developed between the two professional groups. This brings us to the second theme, which focuses on how the collaboration was built and sustained through deliberate relationship-building.

### 3.2. Theme 2: Building Relationships

The second theme centers on the processes through which the interdisciplinary network was established and maintained. Participants emphasized that building relationships—grounded in time, mutual knowledge, and trust—was a foundational element of the collaboration. They described how the network had to evolve in ways that were responsive to the local context rather than following a predetermined model, and how this process involved confronting and dismantling professional prejudices.

For GPs, time is a central concern. While they acknowledged that relationships take time to develop, they also stressed the importance of keeping the process simple and practical given the ongoing time pressure in their work. A GP put it this way: “A manual on how to establish an interdisciplinary network needs to be short if you want to catch our interest. If it’s too long, we won’t read it” (GP 1, Group 1). The metaphor of ‘catching interest’ suggests that involving GPs requires tact and timing—an acknowledgment of how stretched they already are. Developing mutual knowledge as well as mutual trust was another central concern, and both GPs and PMs stressed the importance of physical meetings: “We’re used to thinking that happiness comes from sending an email—but this isn’t something you solve with emails and flyers. We need to meet in person and build mutual understanding. The more you invest, the more you gain” (PM 4, Group 2). GPs described how eye-opening it had been to understand what PMs do in their practice and how they work, and this new kind of knowledge was essential for building relationships. A PM underlined why trust between professions is essential:

They stated that a GP would not want to refer a patient to a PM “who could end up causing more anxiety or distress. That’s why trust and confidence between us are so important” (PM 3, Group 1).

Mutual prejudices were discussed thoroughly in the interviews. For PMs especially, the experience of professional recognition was significant—many had expected to meet resistance but were instead pleasantly surprised. The GPs also acknowledged their own preconceptions. One GP shared that she had initially imagined PMs as highly intellectual and distant: “Extremely clever—not exactly the type you’d just strike up a conversation with” (GP 2, Group 2). The GP found that this presumption did not correspond with reality.

In sum, building relationships was a key aspect, and a combination of local freedom of action and a certain extent of time was essential for developing trusting interdisciplinary collaboration. One of the unforeseen benefits of the collaborative initiative was the disclosure and dismantlement of prejudices. As relationships between GPs and PMs became stronger and more established, participants increasingly oriented their attention toward the ultimate purpose of the collaboration: improving care for patients. This shift in focus is captured in the third theme.

### 3.3. Theme 3: Better Serving the Patients’ Perspective

The third theme reflects participants’ shared conviction that the primary aim of the collaborative initiative was to serve patients better. Discussions therefore focused on patient care and on how the collaboration enabled new ways of addressing existential needs—particularly within an increasingly multi-religious context. Participants further noted that both the timing and extent of patient involvement depended on the vitality and maturity of the local network.

GPs described experiencing frustration with consultations that show no improvement in treatment. One said it this way:

“As a doctor, I sit there with the patient and think: ‘Why aren’t you getting better?’ The patient has been seeing a psychologist, but nothing is changing. And then—bang! This opportunity with the PMs appears” (GP 4, Group 3).

GPs understood that PMs are particularly helpful for patients who are not clinically depressed but are dealing with more existential issues, such as existential life crisis. In these situations, the collaborative initiative is perceived as a relevant option. The GPs elaborate further on this option and reflect on the limits of their professional role and how PMs now complement it. To make their point clear, the GPs describe their practice in generalizing phrases such as measuring, weighing, and testing, and always with a sense of time pressure, while they comprehend the PMs’ practice as an approach without measurements, a softer approach to patients where existential issues can be addressed.

PMs said they joined the collaboration to help spread awareness that contacting a PM is possible in a wide range of situations related to existential issues—and that such contact can be brief and easily arranged:

“Maybe many people have already tried seeing a psychologist, but soul care giver is a different field. We work within another framework, a different lens that has to do with spiritual care” (PM 2, Group 2).

The PMs’ framework and lens towards existential issues is about being with patients in their powerlessness without trying to fix it and to offer spiritual perspectives that can clarify their situation. Both the GPs and the PMs thus embrace the collaborative initiative because of the alternative and multifaced approach to patient care.

Furthermore, PMs reflected on patient contact in a global, multi-religious context. One noted that, in the hospital setting, PMs often talk about existential questions with people from many different religious traditions—and wondered whether this could also happen locally. Would members of other religious groups feel able to contact PMs? The PMs emphasized that this question must be raised within the local community.

A recurring topic under the theme of the patient’s perspective was the extent to which each group had begun involving patients. In Group 1, the participants had decided to first spend time building the interdisciplinary relationship before including patients. This decision led to some frustration and uncertainty over whether the patients would contact the PMs, and whether the potential group of patients was large or small. Despite not having begun patient involvement, the group remained enthusiastic about their meetings, which they described as warm and motivating, rooted in a shared sense of purpose. They maintain focus on patient involvement even though they had not yet done so.

In contrast, Groups 2 and 4 had already begun referring patients to PMs, and the process was working well. It was thus striking that the groups that involved patients from the beginning worked out best, and in the groups without patient involvement there were frustrations. In addition to this observation, Group 5 opted out because they did not want to involve patients, and later this group more or less dissolved. This is an observation we discuss in our critical analysis. It was clear during the analysis of this theme that the GPs and PMs both embraced the collaborative initiative because they experience patients’ need for existential support and these needs could be met by the PMs. The GPs thus experiences the collaboration as a support in patient care. How to address patients with other religious beliefs was a concern, and the PMs were determined to take this up locally.

These experiences of improved patient care and interdisciplinary cooperation naturally led participants to reflect on the broader implications of the initiative, especially regarding the position of PMs within primary care. This forward-looking perspective is captured in the fourth and final theme.

### 3.4. Theme 4: Envisioning PMs’ Role in Primary Care

The fourth theme concerns participants’ reflections on the future role of PMs in primary care and how the collaborative initiative introduces this professional group into primary care in ways that have not previously been formalized.

Participants anticipated a future role of the PM in primary care as one in which they can contribute an important spiritual and humanistic angle that would help not only patients but also GPs and other PMs. The PMs also shared their experience of losing societal authority, along with thoughtful—even hesitant—considerations about their potential future role within primary care. There were also general frustrations about not being a natural and obvious part of patients’ support system. There were discussions of the reasons for this that included PMs having lost authority in society since the 1970s: “Not everyone holds us in high regard. But clearly, that’s not true across the board. This collaboration has shown that we have something valuable to offer—and it’s been meaningful to feel that kind of respect” (PM 1, Group 1).

One GP reflected on upcoming new regulations requiring patient notes to be accessible immediately after consultations [36]:

“Soon patients can read what I write in their journal the very next day. That changes everything—it affects trust between us. I can no longer use the journal as a working tool in the same way. I envy the PMs’ working conditions, and I think we can really complement each other in caring for patients” (GP 5, Group 4).

It is remarkable that this GP thus states that while he estimates a decline in patient confidentiality due to regulations regarding journals, he reflects on the possibility of patients having this confidentiality with PMs.

In this context, the GP mentioned that in parts of Denmark, some PMs now hold consultations in municipal health centers. He found this to be a good idea, as it lends legitimacy to PMs being part of primary care. In response, one PM offered a different view, stressing the value of remaining outside the formal healthcare system:

“It’s part of what defines us as PMs—we’re outside the health care system. We don’t write patient journals; we don’t register visits or ask for health insurance cards or CPR numbers. That’s how it is now, and I think it should stay that way” (PM 7, Group 4).

Among the PMs, stories emerged of professional isolation and a perceived loss of societal authority, and the collaborative initiative was embraced as an opportunity to develop and sharpen their professional identity as well as being integrated in the professional team in patient care. The PMs welcomed the connection with another professional group that also cares for people in crisis. A sense of natural affinity, almost a familiarity, between GPs and PMs arose and the PMs saw it as a natural consequence of both professional groups’ deep engagement in life-defining conditions. As one GP noted, “It is easy to sense and feel that it is two old professions with a vocation, it is something about having a vocation to be where you are and what you are and then do what you can” (GP 5, group 4).

The PMs reflected on how the collaboration aligns with a broader trend toward interdisciplinary cooperation across healthcare settings. PMs experience being invited to talk about the late complications that existential issues can have on rehabilitation—for diabetes patients, cancer patients, and all kinds of patients. They experienced themselves included in both municipal and regional contexts and embraced this as a promising future for both professional groups and patient care.

## 4. Discussion

This study sheds light on the lived experiences of GPs and PMs who established a new local-based collaboration in Denmark. One of the most significant findings in our analysis was that GPs and PMs discovered they could work differently when they met each other in the local collaboration. The GPs realized they did not need to fix everything, especially the things that could not be fixed. They found new courage to remain with patients in their powerlessness, which brought them a sense of relief. They also discovered a new professional group they could recommend to their patients. The PMs discovered that they could play a meaningful and qualified role in patient care. This not only gave them a sense of recognition but also helped develop their professional practices as their pastoral care became more multifaceted, addressing a wider range of themes beyond loss and grief. These discoveries shifted perspectives for both GPs and PMs—what we have termed *reframing*—as they began to see their practice from new angles, with the result of changed practice and approach.

Reframing is a method and a way of seeing life and phenomena. It was first developed as a method in psychotherapy in the 1960s [37] and later adapted in the context of pastoral care [37,38]. Our discussion draws on the work of Donald Capps, former professor of pastoral care, who describes reframing as an art—a hopeful art. It rests on the assumption that people can free themselves from limited perceptions—and, by shifting perspective, discover a broader range of possibilities, both personal and professional. How we understand the meaning of our lives and professional practice depends on the frame through which we perceive them. Changing the frame often leads to a change in perception and, in turn, a change in response, practice, and behavior.

Reframing offers an alternative to the strategy of “more of the same” [38]. For example, one GP described her usual approach: “If we can’t fix something, we try again— schedule another consultation and keep trying.” This reflects a pattern of “more of the same.” Reframing seeks a shift in perspective—one that can alter the interaction in a deeper, more transformative way. To reframe is to see a situation differently, and in doing so, allow the situation itself to change. Our findings show that such a shift in perspective did occur—for both GPs and PMs—and that it changed how they viewed themselves and their professional roles.

This is evident in the way GPs reframed their own self-perception. The shared initiative helped them recognize their habitual approach to certain patients and begin to shift it. Instead of continually trying to fix what could not be fixed, they found value in simply being present with the patient’s struggle and knowing when to refer the patient to a PM.

For their part, the PMs also experienced a reframing of their professional role. Their pastoral care was no longer confined to themes of death and loss—it now extended to a wider range of existential concerns. This challenged and deepened their professionalism, giving them a renewed sense of their contribution to patient care alongside GPs.

The interdisciplinary initiative also reframed the PMs’ role in a multi-religious society. Their practice expanded beyond a Christian context to intentionally include patients from other religious and cultural backgrounds, and to provide existential support across traditions. This is a common experience for chaplains at hospitals, who often provide help across cultures and religions, but it is a relatively new experience for PMs.

It was also found in our study that the collaborative initiative reframed mutual perceptions—prejudices surfaced and were gradually dismantled.

For PMs, the project highlighted a sense of lost authority and legitimacy that they had experienced as a professional group. This led to a reframing of the PM’s role in primary care. Could PMs be formally integrated into the healthcare system, or not? Some GPs in our study advocated including PMs to legitimize their role in patient care. The PMs, however, expressed concerns—particularly around confidentiality and patient trust.

Reframing is a creative method that enables change [37,38]. As our critical analysis has shown, the initiative led to a shift in the perspectives of GPs and PMs despite this never being a direct goal. It was an unexpected gain. However, our observations also underscored that reframing can only occur if the group welcomes the process and embraces what it brings without limiting assumptions.

The participants expressed that relationship-building was a cornerstone in the collaborative initiative. They undertook this task conscientiously and without difficulties, and expressed a sense of familiarity, which one GP suggested had to do with medicine and theology as two practical traditions with a vocation (GP 5, Group 4). This is a rather remarkable statement with even more significance than the GP expressed, because the vocation is entailed in the professional oath. Medical doctors and pastors (along with lawyers) are obliged by their professional oath [39,40] to their fellow humans and not to higher authorities, such as official servants, who are obliged “upwards”, that is to the State. It is not only the two kinds of oath per se but of what lies behind them which is of importance here. The medical doctors’ and pastors’ fundamental obligations to their fellow humans is expressed in two ways: unnegotiable confidentiality and their unnegotiable on-call duty. The confidentiality is fundamental and can only be dispensed to prevent planned criminal acts that are entrusted to the medical doctor or the pastor [41,42,43]. The on-call duty entails that the medical doctor or the pastor must take care of the people who call for medical or pastoral help. It is for this reason that medical doctors automatically react when called on, for example, on airplanes, buses, and trains, and it also means that medical doctors do not send away an acutely sick patient without ensuring that they are taken care of. The same absolute and fundamental obligation is tied to the pastor. They must visit the sick no matter where they are and what they suffer from. They must visit imprisoned criminals and everybody else who asks for pastoral care, regardless of the circumstances or the time of day or night [41]. The fact that both the GP and the PM are bound to an obligation for their fellow human supports the trust required in the interdisciplinary relationship-building.

The need for existential support is well documented [44]. Danish and international research shows that when severe illness and crises strike a person, it often prompts existential concerns [3,14,15]. At the same time, studies from Europe and other Western countries indicate increasing secularization and declining affiliation with organized religion [45,46]. In this context, religion and spirituality tend to be expressed in more individualized ways, which may make it more difficult for GPs to identify and address existential concerns in clinical encounters. Denmark is widely regarded as a highly secularized society [47,48], and similar developments are reported across other countries, suggesting that our findings may also be informative in an international context [49].

Another circumstance that also plays a vital role in relation to trust between the two professional groups is the fact that they form long-term relationships with their patients and parishioners. Continuity of care is of importance here. GPs emphasized in earlier studies that they complete their own course of treatment before recommending that a patient contacts a PM [18]. The expression used by a GP in this study, “course of treatment”, highlights the clinical responsibility while preserving the human dimension. They explained that their long-term relationships with patients help them determine whether such a recommendation is appropriate. It is this ongoing relationship that makes it natural—and comfortable—for the GP to suggest turning to a PM [18]. Along this line, it means that the long-term relation has a stabilizing and reassuring meaning for patients [9,18]. In this context, it is worth noting that the continuity of long-term relationships with a GP is correlated with a longer lifespan and better survival for patients [50,51].

Another fact that supports the collaborative initiative is the new healthcare reform that stressed the need for local healthcare services [52]. Studies from the USA also underline the importance of such initiatives for citizens in local areas, as PMs are professionals who work and often live in the local area and accompany individuals and their families throughout a wide range of life experiences [9]. This correlates with earlier studies where PMs express their professional role as being “*part of the patient journey*” [18]. In this earlier study, we also identified a perception of professional practice that emphasizes continuity and shared care [18]. The professional oath that obliges medical doctors and pastors to their fellow human beings, their confidentiality, their on-call duty, and their presence in patients’ local area all support the value of the collaborative initiative between GPs and PMs for both patient care and their own practice.

It would be valuable to investigate further whether these long-term relationships also support improved existential and spiritual care in primary healthcare in Denmark.

Building on the findings of the present study and as part of the broader action research project, the research group has submitted a request to the Danish National Board of Health to include PMs among the professional groups that GPs may recommend to patients. At the time of writing, the request has not yet been processed. If approved, it would open a new field of practice and research, including questions about implementation, uptake, and outcomes in primary care. In this context, it will also be important to examine funding models for the PM role to support stakeholders interested in adopting similar initiatives. Finally, the research group has planned a study visit to Scotland to learn from existing experience with PMs in primary care and to inform future development of the model.

## 5. Methodological Considerations

We find that the chosen phenomenological hermeneutical FGIs provide useful data in this study. The group setting facilitated open discussions and generated shared reflections that brought about new insights and shifts in perspective regarding participants’ professional identity and practice [28].

The collaboration network was conducted through a participatory design, where the participants themselves volunteered and indirectly initiated the investigation, as they had first contacted Juhl and Vesterdal. The participants were thus positively engaged and co-developers of the project, resulting in a notable absence of negative and critical voices. However, our goal was to develop a manual, not to decide whether to develop one.

The study was small due to the number of participants (13). The first FGI (3 October 2024) had seven participants, and was particularly valuable because it legitimized and confirmed the development of this collaborative initiative. This FGI confirmed the need and value of four further FGIs in each group across the country (January 2025).

The groups were stable and engaged, and participated in each scheduled meeting, whether these were physical, online, or follow-up meetings. We maintained regular and close contact with the groups throughout the process. This resulted in mutual trust and confidentiality, as well as several sessions of supervision. The investigators (Juhl and Vesterdal) thus had insight and knowledge of each group’s process in their local context throughout. It was our experience that a close relationship between the investigators and the participants was necessary to ensure progress and supervision, allowing the participants to maintain their engagement and energy.

The collaborative initiative was developed in a Danish context. When applying the findings to other regions in the world, it is necessary to note that it was developed in relation to the local context and in conformity with the local culture and organizational structure. Our study can inform GPs and PMs around the world about the importance of a local, interprofessional collaboration for the sake of patient care and their own professional development.

In this publication, we have focused on the collaborative initiative and not patients’ experiences. These will be investigated and analyzed in an upcoming publication [19].

We chose a Ricoeur-inspired approach for the study [24,28,32,34,53,54]. A central idea in Ricoeur’s phenomenological hermeneutics is that experiences are not communicated as objective facts, but through personal stories [24,25,26,28,34]. These stories are not copies of reality; they are interpretations of it. Metaphors and figurative language help express what lies beyond everyday language, which is why they are essential in a Ricoeur-inspired analysis [25,26]. The aim of interpretation is to overcome the distance created by time and context, so that what is said in the narrative can emerge clearly [24]. In Ricoeur’s terms, this is “the case in what is said” [24,25]. Through the stories we tell, we reveal how we perceive meaning and significance in our lives—both professionally and personally—that is, how we understand ourselves in the world. The Ricoeur-inspired analysis thus foregrounded their stories and helped us to understand how this collaborative initiative gave meaning to their professional practice. The entire research group participated in interpreting the data and formulating the findings to ensure credibility and trustworthiness.

The project was grounded in mutual respect for difference and a recognition that each professional group brings its own area of expertise. The methodological approach proved suitable because it supported the primary goal and allowed participants to shape and guide the process themselves. However, the approach yielded more than just the design of a manual—it also fostered deep reflection.

## 6. Conclusions

Locally established networks between GPs and Parish Ministers foster a pragmatic, trust-based approach to addressing patients’ existential concerns in primary care. Across five focus group interviews, participants described a reframing of practice: GPs felt licensed not always to “fix,” while PMs broadened their pastoral repertoire beyond bereavement to everyday life crises. Relationship-building and early, appropriate patient inclusion were key to momentum. Future work should assess patient-reported outcomes and explore scalable collaboration models that preserve confidentiality and role clarity—especially in multi-religious communities.

## Data Availability

Data can be made available on reasonable request by contacting Lone Vesterdal. Email: lvesterdal@health.sdu.dk.

## Abbreviations

The following abbreviations are used in this manuscript:

GP	General Practitioner
PM	Parish Minister
